# Pulmonary Enteric Adenocarcinoma: From Diagnostic Attribution and Molecular Biology to Biomarker-Guided Clinical Pharmacology

**DOI:** 10.3390/cancers18213528

**Published:** 2026-11-03

**Authors:** Huei-Yang Hung, Mei-Hsuan Lee, Kuan-Li Wu, Tai-Huang Lee, Chia-Yu Kuo, Cheng-Hao Chuang, Chih-Jen Yang

**Affiliations:** 1 Division of Pulmonary and Critical Care Medicine, Department of Internal Medicine, Kaohsiung Medical University Hospital, Kaohsiung Medical University, Kaohsiung 80708, Taiwan; 1060482@kmuh.org.tw (H.-Y.H.); 970493@kmuh.org.tw (M.-H.L.); klwu@kmu.edu.tw (K.-L.W.); 1080208@kmuh.org.tw (T.-H.L.); cykuo@kmu.edu.tw (C.-Y.K.); 2 Department of Internal Medicine, Kaohsiung Municipal Siaogang Hospital, Kaohsiung 80708, Taiwan; 3 School of Post-Baccalaureate Medicine, College of Medicine, Kaohsiung Medical University, Kaohsiung 80708, Taiwan; 4 Graduate Institute of Medicine, College of Medicine, Kaohsiung Medical University, Kaohsiung 80708, Taiwan

**Keywords:** pulmonary enteric adenocarcinoma, clinical pharmacology, molecular profiling, colorectal metastasis, targeted therapy, immunotherapy, stage IV lung cancer

## Abstract

**Background/Objectives**: Pulmonary enteric adenocarcinoma (PEAC) is a rare lung tumor with intestinal differentiation and marked overlap with metastatic colorectal adenocarcinoma. Misclassification can substantially alter therapeutic decisions. This review integrates contemporary diagnostic, molecular and treatment evidence with an emphasis on clinical pharmacology. **Methods**: In this narrative review, we critically appraised major classification documents, primary clinicopathologic studies, comparative genomic analyses, contemporary PEAC molecular cohorts, pooled outcome reports and landmark biomarker-defined non-small-cell lung cancer (NSCLC) trials published through September 2026; supplementary pharmacology and trial-record verification was performed in October 2026. **Results**: Accurate attribution requires coordinated gastrointestinal evaluation, morphology and a panel of pulmonary and enteric markers; paired sequencing or methylation profiling may help in selected cases. KRAS and TP53 are recurrent alterations, while uncommon EGFR, ERBB2 and kinase-fusion events can inform biomarker-matched treatment. Reported immune features are heterogeneous, and mismatch-repair gene alterations should not be conflated with confirmed microsatellite instability. A pooled analysis of 127 patients (126 published) reported a median overall survival of 14 months in advanced/metastatic disease but did not establish comparative treatment efficacy. Drug interactions, CNS activity and treatment-related lung injury require attention; non-G12C RAS inhibitors remain unvalidated in PEAC. **Conclusions**: Stage IV management should follow molecularly appropriate NSCLC principles while carefully distinguishing directly observed PEAC evidence from extrapolated trial data. Multicenter registries and prospective molecularly annotated studies are urgently needed.

## Simple Summary

Pulmonary enteric adenocarcinoma is a rare form of lung cancer that closely resembles cancer spreading from the intestine. Distinguishing these conditions is essential because their staging and treatments differ. This review explains how clinical evaluation, pathology and molecular testing can be combined to identify the tumor’s origin. We also examine how gene alterations and immune markers may guide drug selection, particularly in patients with metastatic disease. Published treatment reports involve few patients, so responses to a particular medicine should not be mistaken for proven effectiveness in this rare cancer. We propose a practical stage IV treatment framework and highlight the need for international patient registries and biomarker-linked studies.

## 1. Introduction

Pulmonary enteric adenocarcinoma (PEAC) is a rare primary pulmonary malignancy with intestinal morphology. First described by Tsao and Fraser in 1991 [1], it entered the 2011 IASLC/ATS/ERS classification [2] and subsequent WHO classifications [3,4]. The official designation in the fifth edition of WHO Classification of Thoracic Tumours is “enteric-type adenocarcinoma of the lung” [5]. We retain the familiar abbreviation PEAC for continuity with the published literature; it refers to this WHO entity, not to any lung tumor expressing an intestinal marker.

PEAC presents a diagnostic and therapeutic challenge. Its glands, tall columnar cells, pseudostratified nuclei and luminal necrosis resemble colorectal adenocarcinoma metastatic to the lung. However, its molecular profile is heterogeneous and does not simply reproduce colorectal cancer biology [6]. Correct tissue attribution determines staging, molecular testing and drug selection. This review connects integrated diagnosis with biomarker-directed treatment and practical pharmacology while separating direct PEAC observations from broader NSCLC evidence.

The clinical relevance extends beyond diagnostic terminology. A dominant lung mass in a patient with a previous colorectal cancer may represent a new pulmonary primary, whereas a solitary pulmonary lesion can still be a colorectal metastasis. Either assumption can be wrong. Treatment decisions should therefore follow an explicit synthesis of the available evidence, with the level of diagnostic confidence communicated to the treating team. The aim of precision treatment is not merely to find a molecular abnormality but to connect a validated abnormality to the correct disease context, drug, dose and monitoring plan.

## 2. Scope and Evidence Appraisal

This narrative review examines the classification, diagnosis, molecular characteristics and treatment of pulmonary enteric adenocarcinoma (PEAC), covering publications from its first description in 1991 through 30 September 2026. The PubMed/MEDLINE search strategy was retrospectively reconstructed to clarify the review’s scope and facilitate reproducibility. The following search specification combines relevant terminology with publication-date and language restrictions: (“pulmonary enteric adenocarcinoma” [Title/Abstract] OR “pulmonary adenocarcinoma with enteric differentiation” [Title/Abstract] OR ((“enteric-type adenocarcinoma” [Title/Abstract] OR “enteric adenocarcinoma” [Title/Abstract] OR “enteric differentiation” [Title/Abstract]) AND (lung [Title/Abstract] OR pulmonary [Title/Abstract]))) AND (“1991/01/01” [Date—Publication]: “2026/09/30” [Date—Publication]) AND English [Language]. The synthesis prioritizes English-language primary studies of human PEAC with sufficiently detailed pathological, molecular or clinical findings. Classification documents and comparative pathology studies provide the diagnostic framework, while case reports inform uncommon molecular alterations and treatment observations without establishing comparative efficacy. Review articles support reference tracing. Evidence from pivotal non-small-cell lung cancer (NSCLC) trials and regulatory prescribing information is explicitly identified as extrapolation rather than direct PEAC evidence.

Reports of nonpulmonary enteric tumors, confirmed gastrointestinal metastases or cases lacking sufficient diagnostic or outcome information are not used to derive PEAC-specific estimates. Potential cohort overlap is assessed using institutional affiliations, authorship, recruitment periods and patient characteristics. Pooled literature cohorts are considered separately from their constituent reports to avoid double counting. Findings are interpreted in light of sample size, diagnostic certainty, stage distribution, assay methods and treatment setting; no new pooled prevalence or treatment-effect estimates are calculated.

## 3. Classification, Epidemiology and Clinicopathologic Features

The defining features are enteric/colorectal-like architecture in more than 50% of the tumor, evidence of enteric differentiation and clinical exclusion of a gastrointestinal primary [2,3,4,5]. Inamura et al. demonstrated a mixed immunophenotype in seven tumors, with retained CK7 despite intestinal-marker expression [7]. These criteria require assessment of the whole tumor when available; intestinal staining alone does not establish the WHO subtype.

Reported surgical cohorts suggest a prevalence below 1% of pulmonary adenocarcinomas, although changing definitions and referral patterns limit precision. Patients are usually middle-aged or older, with inconsistent sex and smoking associations. In a comparison of 28 PEACs and 92 conventional invasive adenocarcinomas, Zhao et al. associated PEAC with older age, male sex, larger solid tumors and more advanced presentation [8]. These are cohort-specific observations.

Imaging is not diagnostic: PEAC may appear as a spiculated or lobulated solid mass, occasionally with a part-solid or pneumonic pattern. Among 81 resected tumors, 90.1% were pure-solid, 6.2% part-solid and 3.7% pneumonic-type; part-solid lesions occurred only in clinical T1 disease [9]. CEA and CA19-9 elevations can misleadingly suggest gastrointestinal origin [8]. Tall columnar cells typically form irregular glands or cribriform structures with necrotic luminal debris. An admixed conventional pulmonary adenocarcinoma component supports lung origin, but limited sampling may miss it.

## 4. Pathological Diagnosis and Differential Diagnosis

### 4.1. Morphology and Immunophenotype

Morphology alone may not distinguish PEAC from metastatic colorectal carcinoma (MCRC). In nine cases reported by Wang et al., all tumors expressed CK7, whereas CK20, CDX2, MUC2, villin, TTF-1 and Napsin A were variable [10]. Loss of conventional lung markers therefore does not exclude a pulmonary primary.

CK7 supports pulmonary origin but is not exclusive to lung cancer. TTF-1 and Napsin A are helpful when positive but may be absent in PEAC. CDX2, CK20, villin, MUC2 and HNF4α indicate enteric differentiation without establishing tissue of origin [11,12]. A pooled analysis reported sensitivity of 71.3% and specificity of 82% for a CK7+/CDX2+ combination against colorectal adenocarcinoma; the combination is supportive rather than definitive [13].

SATB2 and nuclear β-catenin provide additional discrimination. Matsushima et al. found CK7 in 88% of PEACs, SATB2 in 13% and strong nuclear β-catenin in none, whereas colorectal metastases more often expressed the latter markers [14]. Bian et al. investigated combined CDH17 and SATB2 staining [15]. Assay thresholds, differentiation and comparator selection affect performance; no stain should be treated as an absolute rule.

### 4.2. Excluding Metastatic Colorectal Adenocarcinoma

A diagnosis of PEAC should not be made solely because a lung mass is CK7 positive or because gastrointestinal endoscopy is negative at one time point. The assessment should integrate: (1) dominant lung lesion pattern and radiographic distribution; (2) past history of colorectal or other gastrointestinal cancer; (3) colonoscopy and/or other gastrointestinal evaluation when clinically indicated; (4) morphology; (5) a pulmonary-plus-enteric IHC panel; and (6) molecular comparison when both pulmonary and colorectal tumors are available.

Gastrointestinal evaluation should be proportionate to the presentation. Prior endoscopy and pathology reports should be reviewed, including the timing and completeness of investigations. A negative examination reduces the likelihood of an identifiable gastrointestinal primary but cannot establish pulmonary lineage by itself. When there is a known colorectal tumor, side-by-side morphological review and comparison of the complete immunophenotype are more informative than a single positive stain. Discordance should trigger reassessment of both specimens rather than selective acceptance of the result that fits the presumed diagnosis. The final report should document the evidence supporting origin and any unresolved limitation.

A first-line panel comprises CK7, TTF-1, Napsin A, CK20 and CDX2. CDX2 and CK20 establish an enteric phenotype but lack organ specificity. In contrast, diffuse SATB2 and strong nuclear β-catenin favor colorectal origin and weigh against PEAC, particularly when lung markers are absent. Their negativity does not exclude colorectal carcinoma, and occasional PEACs express SATB2 [14,15]. CDH17 and HNF4α are ancillary markers, not independent tissue-of-origin tests. Clinical history and paired specimens remain essential when staining is discordant.

Paired sequencing can support or challenge a common origin when both gastrointestinal and pulmonary tumors are available, although a shared common driver alone does not establish clonality. Zuo et al. demonstrated distinct genomic and methylation patterns between PEAC and lung metastatic colorectal cancer [16]. Their methylation classifier is promising for difficult cases, but external validation and clinical assay standardization are required before routine adoption.

For a small core biopsy, recommended descriptive wording is “Adenocarcinoma with enteric differentiation.” An accompanying comment can state: “The sampled tumor shows enteric morphology and immunophenotype. This biopsy cannot establish whether enteric architecture exceeds 50% of the entire tumor. Correlation with clinical history, imaging and appropriate gastrointestinal evaluation is required to distinguish a pulmonary primary from metastatic gastrointestinal adenocarcinoma.” When the integrated evidence supports lung origin, “favor pulmonary primary” may be added; definitive WHO subtyping should remain qualified until adequate sampling supports it. This is practical reporting guidance, not a mandatory WHO reporting template. Conventional pulmonary adenocarcinoma with focal enteric differentiation, invasive mucinous or colloid adenocarcinoma and other gastrointestinal metastases remain differential diagnoses.

A further distinction is the percentage of tumor sampled versus the percentage of the entire lesion. Even a core composed entirely of enteric glands does not demonstrate that more than half of the whole tumor has this architecture. Conversely, a small conventional pulmonary component on a core may support lung origin without resolving the overall enteric percentage. The report should identify the specimen type and avoid presenting the observed biopsy proportion as a whole-tumor measurement. Preserving unstained sections or a suitable block for molecular testing is especially important when additional immunostains could exhaust limited material.

Table 1 consolidates the diagnostic workflow and the relative value of individual immunohistochemical markers.

## 5. Molecular Landscape and Biological Evolution

Molecular estimates vary with geography, stage, diagnostic criteria and assay coverage. Mutation, amplification and fusion are not interchangeable categories. Table 2 therefore supplies cohort-specific numerators and denominators alongside a defined lung adenocarcinoma comparator, rather than a pooled “NSCLC frequency.” Lack of a reliable PEAC denominator is explicitly distinguished from absence of an alteration.

Comparison also requires attention to the denominator. A study may include many patients but sequence only the subset with adequate tissue; frequencies should use the tested denominator and describe selection. Results from resection cohorts may underrepresent patients with aggressive unresectable disease, while molecular referral cohorts may overrepresent unusual or actionable cases. These differences can produce apparently conflicting proportions without proving biological disagreement. Reporting exact counts, mutation classes and testing methods is therefore more useful than presenting a single summary percentage with misleading precision. The same principles apply to PD-L1 and MMR analyses.

KRAS is recurrent, but its frequency is not uniformly high. Zuo et al. detected mutations in 6/32 tumors, whereas Liu et al. found 8/17 [16,17]. In the latter cohort, G12D and G12V each accounted for 3/8 KRAS-mutant tumors, with G12A and G12C each accounting for 1/8. These small denominators limit population-level inference but make allele-level reporting essential: G12C-directed drugs cannot be substituted for therapies targeting G12D or G12V.

EGFR mutations remain clinically relevant despite a lower prevalence than in many East Asian lung adenocarcinoma series. Liu et al. reported selected patients benefiting from osimertinib [17]. ERBB2 mutations were present in 4/32 tumors in Zuo et al., including exon 20 insertions and missense variants [16]. A mutation must be distinguished from amplification or protein overexpression when applying a HER2-directed indication. ALK-related events also require confirmation of a therapeutically relevant rearrangement rather than an unspecified sequence change.

For conventional lung adenocarcinoma, TCGA provides a transparent comparator of 230 resected, untreated tumors: KRAS, EGFR and TP53 mutations occurred in approximately 33%, 14% and 46%, respectively [18]. This cohort is not geographically or stage matched to PEAC studies. Broad gene-level mutation rates, especially for BRAF or MET, must not be equated with BRAF V600E or MET exon 14 skipping prevalence. For rare fusions and other actionable PEAC alterations, current data do not support stable frequencies.

Enteric morphology does not imply colorectal oncogenesis. APC enrichment in colorectal metastases and distinct methylation profiles support biological separation [16]. Pulmonary lineage plasticity is a plausible explanation for intestinal differentiation, but longitudinal studies are needed to distinguish true transdifferentiation from selection of pre-existing tumor components.

Immune findings are inconsistent across small cohorts. Earlier reports described elevated mutation burden or MMR-pathway alterations [13], whereas Liu et al. reported PD-L1 positivity in 3/17 tumors and no MSI-high cases [17]. The available abstract does not specify the PD-L1 scoring cutoff, so this proportion is not relabeled as TPS ≥ 1%. Here, tumor proportion score (TPS) denotes the percentage of viable tumor cells with membranous PD-L1 staining; it is distinct from the combined positive score, which also includes positive immune cells. Clinical interpretation requires the assay, scoring system and threshold. In Yang et al., all seven tested tumors retained MMR proteins and were PD-L1-negative [19]. Retained MMR staining supports proficient MMR but is not itself a molecular MSI assay. Likewise, an MMR gene variant does not establish functional dMMR. These biomarkers should be evaluated separately, without assigning a universal immune phenotype to PEAC.

**Figure 1:**
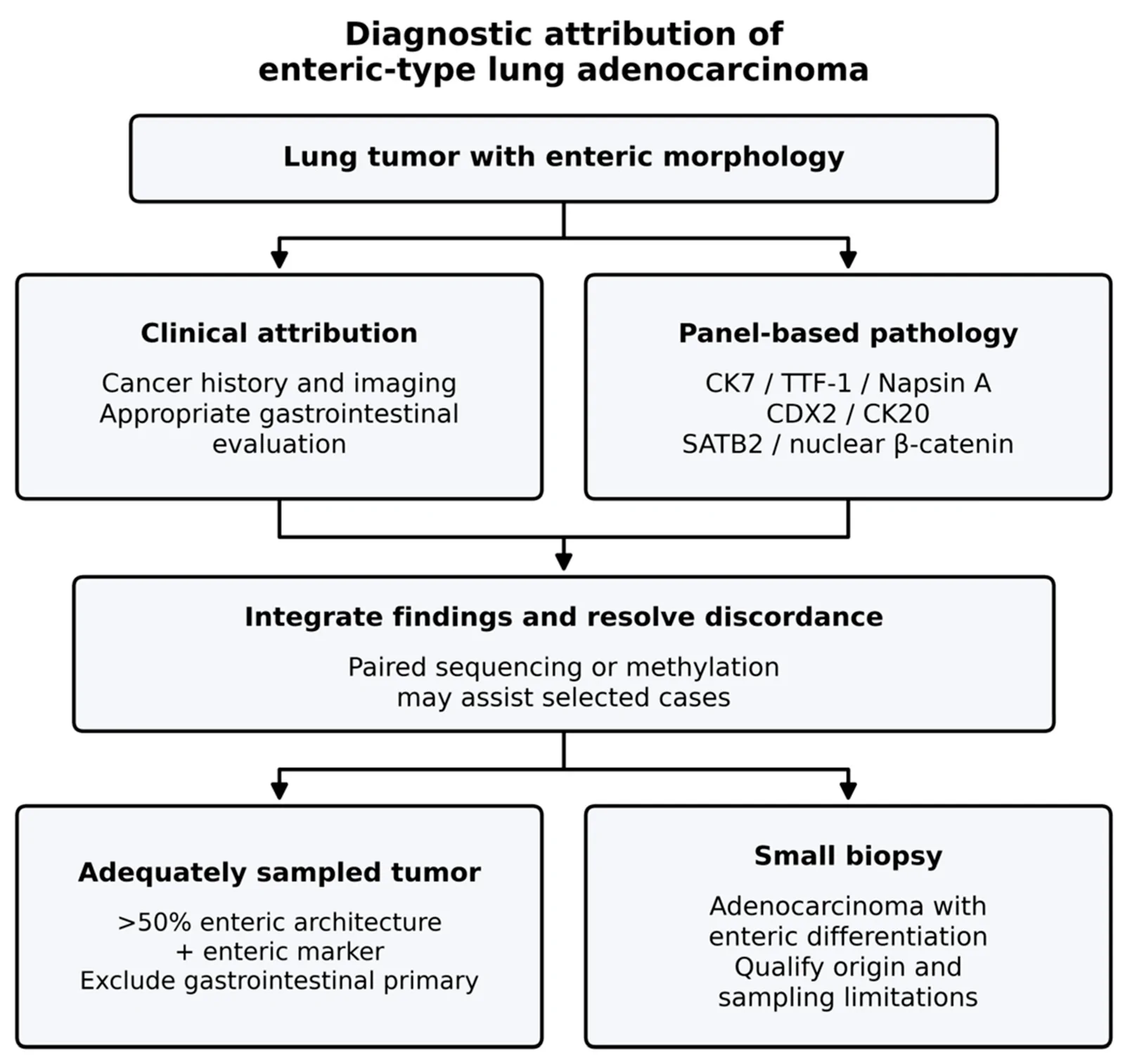
Integrated diagnostic attribution of enteric-type adenocarcinoma of the lung. Enteric morphology initiates combined clinical and immunohistochemical assessment. Small-biopsy wording remains qualified because whole-tumor architecture cannot be established. CDX2/CK20 indicate differentiation, whereas SATB2 and nuclear β-catenin contribute to tissue attribution [2,3,4,5,14,15,16]. IHC, immunohistochemistry; GI, gastrointestinal.

Table 2 compares selected driver frequencies with a defined conventional lung adenocarcinoma cohort and summarizes immune biomarker limitations. Figure 1 outlines the diagnostic workflow.

## 6. Clinical Pharmacology and Treatment Strategies

### 6.1. Localized and Locally Advanced Disease

There are no randomized PEAC-specific treatment data. Operable localized disease is managed with pulmonary resection and appropriate nodal assessment according to NSCLC principles. Tumor size, location and pulmonary reserve guide the extent of surgery. Retrospective studies support stage as a major prognostic determinant but cannot establish superiority of a specific operation or adjuvant regimen [20].

Adjuvant treatment should follow stage, pathological risk and validated molecular indications. Enteric morphology alone does not justify colorectal-type chemotherapy. Unresectable stage III disease warrants consideration of definitive chemoradiotherapy and biomarker-appropriate consolidation using NSCLC evidence. Whether PEAC differs in radiosensitivity is unknown.

### 6.2. Advanced Disease: Chemotherapy, Targeted Therapy and Immunotherapy

Published PEAC treatment reports describe platinum combinations with pemetrexed, taxanes or gemcitabine, with heterogeneous outcomes [21,22,23]. Fluoropyrimidine/oxaliplatin- and irinotecan-based colorectal regimens have also been tried. Isolated nonresponses do not prove ineffectiveness, but there is no comparative evidence supporting their routine preference over lung cancer regimens [24]. Tissue origin, rather than enteric staining alone, should determine the treatment framework.

Broad DNA profiling with RNA-based fusion assessment where feasible is preferable to restricted testing. Selected EGFR-mutant and ALK-rearranged PEAC patients have benefited from matched inhibitors [17], supporting molecular testing without establishing PEAC-specific response rates. Other validated NSCLC targets should be interpreted by exact variant, prior therapy and local indication. A pathway alteration labeled “potentially actionable” in sequencing research is not synonymous with an approved predictive biomarker.

Testing should be completed early enough to inform the initial treatment decision whenever clinically feasible. A negative limited hotspot panel does not establish the absence of all actionable alterations, and DNA-only assays may have different sensitivity for individual fusions than RNA-based methods. Variant annotation should distinguish a pathogenic driver, a resistance alteration and a variant of uncertain significance. Where a finding is unexpected for the phenotype, laboratory review and correlation with tumor content can help avoid misinterpretation. The molecular report should specify the exact alteration used to justify treatment, rather than simply labeling a tumor “EGFR-positive” or “HER2-positive.”

Checkpoint inhibition is selected using driver status, validated PD-L1 testing, disease burden and contraindications. Fassi et al. reported dMMR in 3/20 selectively tested published cases; this is not a population prevalence [25]. Case-level chemoimmunotherapy benefit in Liu et al. is encouraging, but neither high TMB nor a suggested immune-cold phenotype has been prospectively validated as a PEAC-specific predictor [13,17,19].

A high PD-L1 result should not bypass assessment for an actionable driver, and a low result does not by itself establish futility of chemotherapy plus immunotherapy. The relevant question is whether the proposed regimen has evidence in the corresponding molecular and clinical NSCLC setting. In PEAC, treatment selection should acknowledge uncertainty while retaining standard contraindication assessment and patient-centered discussion. Published exceptional responders are useful signals for research, but they do not identify the probability that a new patient will respond. Negative and short-lived responses deserve equal capture in future studies to reduce publication bias.

For KRAS G12D disease, early clinical development includes the selective RAS(ON) inhibitor zoldonrasib (RMC-9805; NCT06040541), studied alone and with RMC-6236 [26]. Daraxonrasib (RMC-6236; NCT05379985) is a multi-selective RAS(ON) inhibitor studied across specified RAS-mutant solid tumors, providing a development route relevant to G12D and G12V [27]. “Pan-KRAS” and “multi-RAS” should not be used interchangeably: target isoform, allele and nucleotide state differ. These trials do not establish efficacy in PEAC or justify off-label extrapolation from pancreatic cancer. Eligibility, recruitment and emerging results require verification at referral. No PEAC-specific efficacy estimate is available for non-G12C inhibitors.

### 6.3. Pharmacokinetics, Drug Interactions and CNS Activity

Medication reconciliation is part of biomarker-matched prescribing. Many oral TKIs are affected by CYP3A-mediated metabolism, but the magnitude and management of interactions are agent-specific. Strong inducers such as rifampin, carbamazepine and phenytoin can reduce exposure; strong inhibitors may increase exposure and toxicity. Lorlatinib is a particularly important example: strong CYP3A inducers are contraindicated because of both reduced exposure and serious hepatotoxicity, while unavoidable strong inhibitors require label-directed dose adjustment [28]. Supplements, antiseizure drugs and supportive medicines should therefore be reviewed before treatment and at each transition. Class-wide empirical dose changes are inappropriate.

A practical interaction review should identify the interacting drug, the expected direction of exposure change, the clinical consequence and the permitted management under the specific label. Substituting an interacting concomitant medicine may be preferable to changing an effective anticancer regimen, but this depends on the alternative’s suitability. Starting or stopping an inducer can change exposure over time, so a medication list checked only at baseline is insufficient. New toxicity after a medication change and unexpected loss of disease control should both prompt renewed review. Evidence of an interaction should be distinguished from assumptions based only on shared metabolism.

CNS disease introduces a second pharmacologic constraint. Drug delivery across the blood–brain barrier, transporter efflux and intracranial target inhibition differ among agents; systemic response does not ensure CNS control. Osimertinib has demonstrated intracranial activity in the FLAURA CNS analysis [29], and lorlatinib was developed for CNS-active ALK inhibition [30]. These data support choosing a CNS-active matched agent when appropriate but are extrapolated from biomarker-defined NSCLC rather than PEAC. Neurological symptoms, lesion size and mass effect still determine the need for local therapy. CNS progression should prompt assessment of adherence, interactions, extracranial control and resistance, rather than automatic dose escalation.

CNS efficacy is also an endpoint issue. Trials differ in whether they enroll untreated, stable or symptomatic brain metastases and in whether intracranial lesions are measurable. Consequently, a systemic response rate cannot be compared directly with an intracranial response rate from another population. In a PEAC case report, the timing of radiotherapy, baseline neurological status and concurrent corticosteroid exposure should be documented before attributing intracranial improvement entirely to a targeted drug. Prospective collection of brain imaging and site-specific progression would help clarify whether CNS failure reflects inadequate exposure or biological resistance.

Exposure and tolerability also depend on organ function and regimen composition. For trastuzumab deruxtecan, DESTINY-Lung02 illustrates the clinical dose–toxicity tradeoff: adjudicated drug-related ILD occurred in 12.9% at 5.4 mg/kg and 28.0% at 6.4 mg/kg every three weeks; grade ≥ 3 drug-related adverse events occurred in 38.6% and 58.0%, respectively [31]. These are dose-group observations, not a patient-level exposure threshold. The NSCLC dose is 5.4 mg/kg every three weeks in the cited indication [32]. New respiratory symptoms require prompt investigation and treatment interruption; confirmed grade ≥ 2 ILD requires permanent discontinuation under the prescribing information. PEAC-specific susceptibility is unknown. Baseline lung disease, prior pulmonary toxicity and competing infection or progression complicate attribution.

**Figure 2:**
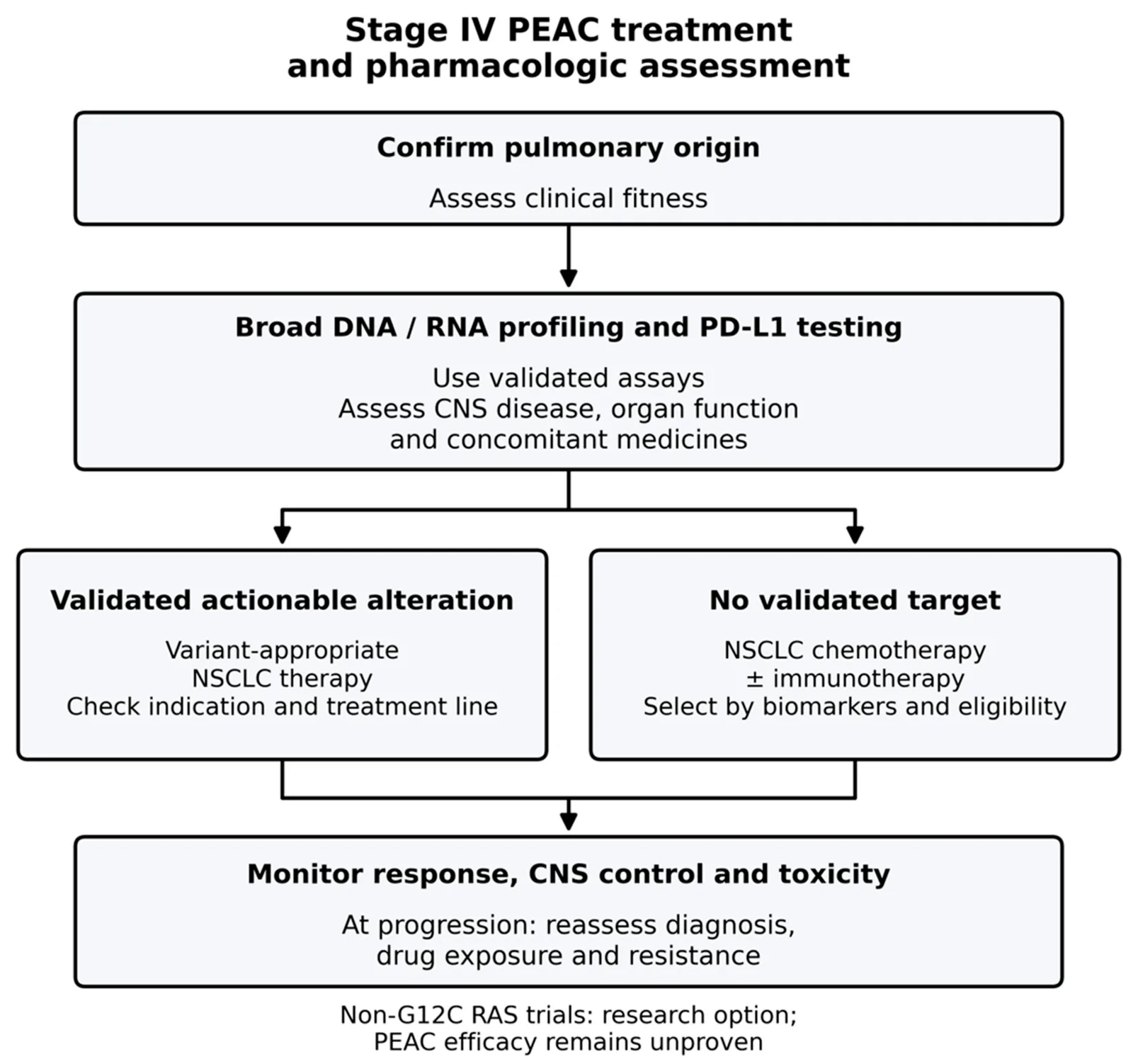
Biomarker-guided stage IV PEAC treatment framework. This is an extrapolated NSCLC framework, not a validated PEAC algorithm. Genotype, CNS disease, interactions, organ function and adverse-event risk inform drug selection. Non-G12C RAS trials are investigational for PEAC [17,26,27,28,29,30,31,32,33,34,35,36,37,38,39,40,41,42,43,44,45,46]. CNS, central nervous system; NGS, next-generation sequencing; TPS, tumor proportion score.

A practical stage IV sequence is to confirm pulmonary origin, obtain broad molecular testing and PD-L1 assessment and select genotype-matched treatment when a validated indication exists. Negative plasma testing should be followed by tissue testing when feasible. Without a target, nonsquamous NSCLC chemotherapy with or without checkpoint blockade is selected according to clinical eligibility [30,33,34,35,36,37,38,39,40,41,42,43,44,45,46]. Figure 2 summarizes this approach; Table 3 distinguishes direct observations from extrapolation. Toxicity monitoring should be drug-specific, including hepatic and metabolic monitoring for lorlatinib, cardiac assessment when indicated and pneumonitis surveillance for agents carrying ILD risk. No PEAC-specific dose modification is established.

### 6.4. Response, Resistance, Toxicity and Interpretation of Survival

Response assessment should report objective response, duration, progression-free survival, overall survival and treatment-limiting toxicity. Repeat tissue or plasma profiling at progression can reveal on-target resistance or bypass pathways; tissue is especially useful when histological change is suspected. Negative plasma findings do not exclude resistance mechanisms. Evidence for a standard PEAC treatment sequence is absent. Dose interruption, reduction and discontinuation should be recorded separately from biological progression, because intolerance can shorten treatment duration without demonstrating lack of activity.

Toxicity assessment should include severity, timing, causality and reversibility. Pulmonary symptoms during treatment are particularly difficult to interpret because infection, tumor progression, radiation injury and drug-induced pneumonitis can overlap. Investigations and management should follow the suspected agent and clinical severity rather than presuming that every new opacity represents an immune-related event. Likewise, stopping treatment for toxicity should not be merged with progression in an efficacy endpoint without explanation. Treatment duration and progression-free survival answer different questions; future PEAC studies should define both clearly and report subsequent treatment when interpreting overall survival.

### 6.5. Evidence Limitations and Trial Priorities

Single-patient responses establish plausibility, not comparative effectiveness. Genotype, stage, treatment line, access and selective publication confound comparisons between chemotherapy, targeted therapy and immunotherapy. Prospective registries should capture dose intensity, interacting medicines, CNS disease, organ function and adverse events alongside molecular and survival outcomes. Basket trials should prespecify PEAC strata and central pathology review whenever feasible.

For example, a registry should avoid counting a patient described first in a case report and subsequently in an institutional series as two independent observations. Recruitment periods and institutions can identify potential overlap, but matching by age, sex and treatment sequence may still be inconclusive. Unresolved duplication should be stated, and quantitative pooling should be avoided when individual independence cannot be established. This review therefore presents selected cohorts separately and does not derive an overall PEAC treatment-response rate.

## 7. Prognosis and Patterns of Recurrence

Stage is the most consistent prognostic determinant, although estimates are imprecise. Wang et al. reported median OS of 38 months in a 26-patient institutional cohort and 28 months in a separate 20-patient SEER cohort [20]. In 81 resected patients, Du et al. identified stage III as an independent recurrence risk factor and pathological nodal involvement as a predictor of poorer survival [9]. These selected populations cannot serve as universal prognostic benchmarks.

Recurrence may be intrapulmonary, nodal or distant; intrapulmonary metastasis predominated in the Du surgical series [9]. No robust evidence establishes a PEAC-specific organ tropism or molecular recurrence marker independent of stage. Fassi et al. pooled 127 patients, including 126 published cases, and reported median OS of 56 months in early-stage and 14 months in advanced/metastatic disease [25]. These descriptive estimates cannot be attributed to a particular drug or compared directly with modern biomarker-selected trials.

Reported outcomes differ substantially by stage, clinical selection and data source; Table 4 summarizes noninterchangeable estimates.

## 8. Knowledge Gaps and Future Directions

Progress requires shared diagnostic standards and prospective data collection. Central review using WHO terminology, adequate morphological sampling and documented gastrointestinal evaluation would reduce misclassification. A registry should record whether PEAC was diagnosed on resection or only suspected on biopsy, preserving diagnostic uncertainty rather than treating both as equivalent.

Integrated DNA, RNA, methylation and immune profiling may identify reproducible biological subgroups. Methylation-based attribution remains a promising research adjunct [16] but must be tested in real-world equivocal specimens, including small biopsies and unusual gastrointestinal metastases. Independent validation should report failure rates as well as classification accuracy.

Treatment registries should harmonize stage, genotype, PD-L1 assay/TPS, functional MMR status, molecular MSI results, treatment sequence and sites of progression. Linking drug exposure and toxicity to outcomes would strengthen pharmacologic interpretation. For immunotherapy, separate reporting of tumor-cell PD-L1, immune infiltration and TMB is preferable to grouping these distinct measures as “immunogenicity.”

A feasible prospective registry could link a diagnostic dataset to a longitudinal treatment dataset. The first would include specimen type, morphological proportions when assessable, immunostain results, gastrointestinal workup and central review. The second would record molecular testing dates, actual drug exposure, dose changes, concomitant medicines, imaging assessments and reasons for discontinuation. Recording successive lines of therapy would allow hypotheses about resistance and tolerability to be examined without treating all systemic exposure as a single category. Such a design would still be observational, but transparent eligibility and standardized follow-up would improve substantially on selectively published cases.

Pathologists and oncologists should preserve tissue for molecular testing while resolving origin. Discordant clinical, immunohistochemical or molecular findings merit multidisciplinary review. International collaboration can make rare, rigorously annotated cases useful without overinterpreting their numerical precision.

## 9. Conclusions

Enteric-type adenocarcinoma of the lung requires integrated tissue attribution, not reliance on intestinal markers alone. Limited biopsies warrant qualified descriptive reporting. Molecular profiling can identify actionable variants, but frequencies are cohort-dependent and most treatment evidence is extrapolated from NSCLC. Drug interactions, CNS activity and exposure-related toxicity should inform prescribing alongside genotype. Non-G12C RAS-directed trials provide a research opportunity rather than an established PEAC standard. Prospective, centrally reviewed cohorts are needed to connect diagnosis, drug exposure and meaningful clinical outcomes.

## Figures and Tables

**Table 1:** Integrated diagnostic framework.

Assessment	Findings Supporting PEAC	Findings Favoring Metastatic CRC/Key Limitation	Refs.
Morphology	Predominant (>50%) enteric glands, columnar cells, cribriform pattern; possible admixed conventional lung component.	Overlap with CRC is considerable; the >50% threshold is unreliable in a small biopsy.	[2,3,4,7]
Clinical/radiology	Dominant pulmonary lesion and no established gastrointestinal primary after appropriate evaluation.	Known GI tumor, tissue concordance and clinical metastatic pattern require reassessment; negative endoscopy alone does not prove PEAC.	[8,10]
CK7	Frequently positive in PEAC.	Usually negative in CRC but positive CRC metastases exist; interpret with the full panel.	[7,10,13,14]
TTF-1/Napsin A	Expression, when present, supports pulmonary origin.	Frequently absent in PEAC; absence does not establish metastatic CRC.	[7,10,14]
CDX2/CK20/villin/MUC2	Support an enteric differentiation phenotype.	Also expressed by CRC; none independently establishes organ of origin.	[10,13,14]
SATB2	Often absent or focal in reported PEAC series.	Diffuse/strong SATB2 favors colorectal origin but is not perfectly specific.	[14,15]
β-catenin	Strong nuclear staining was uncommon in a small PEAC comparator series.	Strong nuclear localization adds support for CRC, particularly with diffuse SATB2.	[14]
CDH17/HNF4α	May be useful as ancillary markers within a broader panel.	CRC commonly expresses these markers; CDH17 with SATB2 may aid discrimination. Neither CDH17 nor HNF4α independently establishes origin.	[11,12,15]
Paired molecular/methylation	A discordant genotype or characteristic methylation signature can inform difficult tissue attribution.	Experimental adjunct, not a substitute for clinical and pathological exclusion of GI cancer.	[16]

Abbreviations: CRC, colorectal carcinoma; GI, gastrointestinal; IHC, immunohistochemistry; PEAC, pulmonary enteric adenocarcinoma. Patterns summarize small comparative series and are not absolute diagnostic rules. Marker performance depends on assay and cutoff. References are provided per row.

**Table 2:** Driver frequencies and immune biomarker interpretation.

Alteration	PEAC Cohorts	Conventional LUAD Comparator	Interpretation
KRAS mutation	6/32 (18.8%) [16]; 8/17 (47.1%) [17]; 21/68 (30.9%) [9]	33% in TCGA (*n* = 230) [18]	Cohort-dependent. In [17], G12D and G12V each 3/8 KRAS-mutant tumors; G12C 1/8.
EGFR mutation	5/32 (15.6%) [16]; 1/17 (5.9%) in molecular subset [17]; 6/68 (8.8%) [9]	14% in TCGA [18]	Not a geographically matched comparison; exact variant determines actionability.
ERBB2 mutation	4/32 (12.5%): two exon 20 insertions and two missense variants [16]	4% in the tLUAD comparator used by Zuo et al. [16]	Mutation, amplification and IHC expression are distinct categories.
ALK alteration	2/17 (11.8%) ALK-related events reported [17]	Not quantified here	Do not interpret unspecified ALK events as a confirmed fusion prevalence.
TP53 mutation	12/17 (70.6%) [17]	46% in TCGA [18]	Co-alteration; no established PEAC-specific matched therapy.
BRAF V600E; MET exon 14; RET, ROS1, NTRK fusions	Insufficient consistent PEAC denominators	No pooled comparator assigned	Broad gene mutation rates are not actionable variant frequencies; include in comprehensive testing.
PD-L1	3/17 positive [17]; 0/7 positive [19]	Not compared	Original scoring and cutoffs must be verified; positivity is not automatically TPS ≥ 1%.
MMR/MSI	No MSI-H in 17 tested [17]; retained MMR proteins in 7 [19]	Not compared	Retained MMR IHC, molecular MSS and an MMR gene variant are different measurements.

Abbreviations: LUAD, lung adenocarcinoma; TCGA, The Cancer Genome Atlas; TPS, tumor proportion score; tLUAD, typical lung adenocarcinoma; IHC, immunohistochemistry; MMR, mismatch repair; MSI-H, microsatellite instability-high. Percentages describe separate tested cohorts, not pooled prevalence. TCGA comprises 230 resected untreated tumors and is not matched to PEAC cohorts. The ERBB2 comparator comes from [16], not TCGA. Missing estimates mean insufficient comparable data, not zero prevalence.

**Table 3:** Treatment evidence and pharmacologic considerations.

Setting/Biomarker	Treatment Framework	Evidence and Pharmacologic Caveat	Refs.
Resectable I–II	Surgical resection and nodal assessment; consider stage/molecularly indicated adjuvant therapy.	PEAC evidence is observational; enteric morphology alone does not justify CRC adjuvant chemotherapy.	[20,25]
Unresectable stage III	Definitive chemoradiotherapy and suitable NSCLC-based consolidation.	No PEAC-specific trials; follow current molecular and immunotherapy eligibility.	[21,22,23]
Stage IV, EGFR-mutant	Variant-appropriate EGFR TKI (e.g., osimertinib for eligible sensitizing variants).	Selected PEAC responses; pivotal efficacy and CNS data derive from broader EGFR-mutant NSCLC. Monitor ILD and cardiac risk.	[17,33,34]
Stage IV, ALK/ROS1/RET/METex14/BRAF V600E/NTRK	Use genotype-matched approved or guideline-supported NSCLC therapy.	ALK PEAC case-level benefit; other PEAC data sparse. Confirm exact alteration and treatment line.	[17,30,35,36,37,38,39]
Stage IV, HER2 activating mutation	Consider eligible HER2-directed agents where approved.	Broader NSCLC evidence for trastuzumab deruxtecan; monitor drug-related ILD. Not all HER2 alterations are equivalent.	[17,40]
Stage IV, KRAS G12C	A KRAS G12C inhibitor in the indicated line for eligible patients.	G12C evidence derives from NSCLC. G12D/G12V are distinct; investigational RAS-directed trials are discussed in Section 6.2.	[17,41,42]
Stage IV, driver-negative	Platinum/pemetrexed with or without PD-1/PD-L1 blockade per current nonsquamous NSCLC practice.	NSCLC extrapolation; select by PD-L1, clinical fitness and contraindications. PEAC drug-specific effect is unquantified.	[17,43,44,45]
Stage IV, confirmed MSI-H/dMMR	Consider immune checkpoint therapy if indicated under applicable tumor-agnostic/local rules.	MSI-H appears uncommon in recent small PEAC cohorts, although dMMR was reported in 3 of 20 tested pooled published cases. MSI-H CRC trial data are context, not direct PEAC efficacy.	[17,19,25,46]
Progression/resistance	Clinical reassessment, repeat tissue or plasma molecular testing and trial enrollment.	No validated PEAC drug sequence; assess toxicity, co-mutations, CNS disease and access.	[17,21,22,23]
Pharmacology across genotypes	Review CYP3A interactions, CNS activity and exposure-related toxicity.	Lorlatinib interaction precautions; T-DXd dose-related ILD findings derive from labeling/NSCLC, not PEAC.	[28,29,31,32]

Abbreviations: CRC, colorectal cancer; dMMR, deficient mismatch repair; ILD, interstitial lung disease; MSI-H, microsatellite instability-high; NSCLC, non-small-cell lung cancer; PD-1, programmed death 1; PD-L1, programmed death ligand 1; TKI, tyrosine kinase inhibitor. PEAC-specific support is largely retrospective or case-level; cited NSCLC trials do not establish PEAC-specific response rates. Availability and treatment line vary by jurisdiction.

**Table 4:** PEAC cohorts and outcome interpretation.

Study/Cohort	Population	Selected Results	Interpretive Limitation
Fassi et al. (2023) [25]	127 patients: 126 published cases plus 1 institutional case.	Median OS: early stage, 56.0 months; advanced/metastatic, 14.0 months. Postoperative median DFS, 24 months.	Published-case pooling, mixed era, selection/publication bias; not regimen-specific efficacy.
Wang et al. (2023) [20]	Single-center cohort *n* = 26; independent SEER cohort *n* = 20.	Institutional median OS, 38 months; median PFS, 8 months; stage III–IV median PFS, 6 months. SEER median OS, 28 months.	Mixed-stage retrospective populations. Cross-cohort median OS values should not be directly compared.
Du et al. (2026) [9]	Single-center resected cohort, *n* = 81 (stages I–III; advanced-stage cases excluded).	5-year OS: 81% (p-stage I), 70.1% (II), 45% (III). KRAS 21/68 (30.9%), EGFR 6/68 (8.8%). Intrapulmonary metastasis was the predominant recurrence pattern.	Surgical series only; 90.1% pure-solid on CT. Findings do not apply to stage IV disease.
Liu et al. (2023) [17]	24 PEAC patients; *n* = 17 with tumor DNA/RNA and immune assays.	TP53 12/17 (70.6%); KRAS 8/17 (47.1%); selected case-level targeted and chemoimmunotherapy benefit.	Small molecular subset, heterogeneous treatment lines; no comparative drug-specific response estimate.
Yang et al. (2025) [19]	10 PEAC patients; *n* = 7 evaluable for immune studies.	Seven tested tumors retained MMR proteins and were PD-L1-negative; low immune-cell infiltration.	Small selected cohort. Retained MMR staining is not a molecular MSI result; no validated prediction of treatment response.

Abbreviations: DFS, disease-free survival; MSS, microsatellite stable; OS, overall survival; PD-L1, programmed death ligand 1; PEAC, pulmonary enteric adenocarcinoma; PFS, progression-free survival; SEER, Surveillance, Epidemiology and End Results. Estimates come from separate observational studies with different stage distributions and selection mechanisms; reported medians are descriptive and not head-to-head treatment comparisons.

## Data Availability

This narrative review was conducted through a structured literature search primarily using PubMed/MEDLINE. No new data were created in this study. Data sharing is not applicable to this article.
